# Editorial: Outstanding advances in veterinary diagnostic ultrasonography: novel milestones in disease detection, prediction, and treatment

**DOI:** 10.3389/fvets.2025.1675622

**Published:** 2025-09-16

**Authors:** Rio Hayashi, Hussein M. El-Husseiny, Ryou Tanaka

**Affiliations:** ^1^Veterinary Teaching Hospital, Tokyo University of Agriculture and Technology, Tokyo, Japan; ^2^Institute of Global Innovation Research, Tokyo University of Agriculture and Technology, Fuchu-shi, Tokyo, Japan; ^3^Department of Surgery, Anesthesiology, and Radiology, Faculty of Veterinary Medicine, Benha University, Toukh, Egypt; ^4^Laboratory of Veterinary Physiology, Department of Veterinary Medicine, Tokyo University of Agriculture and Technology, Tokyo, Japan

**Keywords:** ultrasonography, veterinary practice, diagnostic imaging, echocardiography, IVPG, 2DTT, VFM

**Figure 1:**
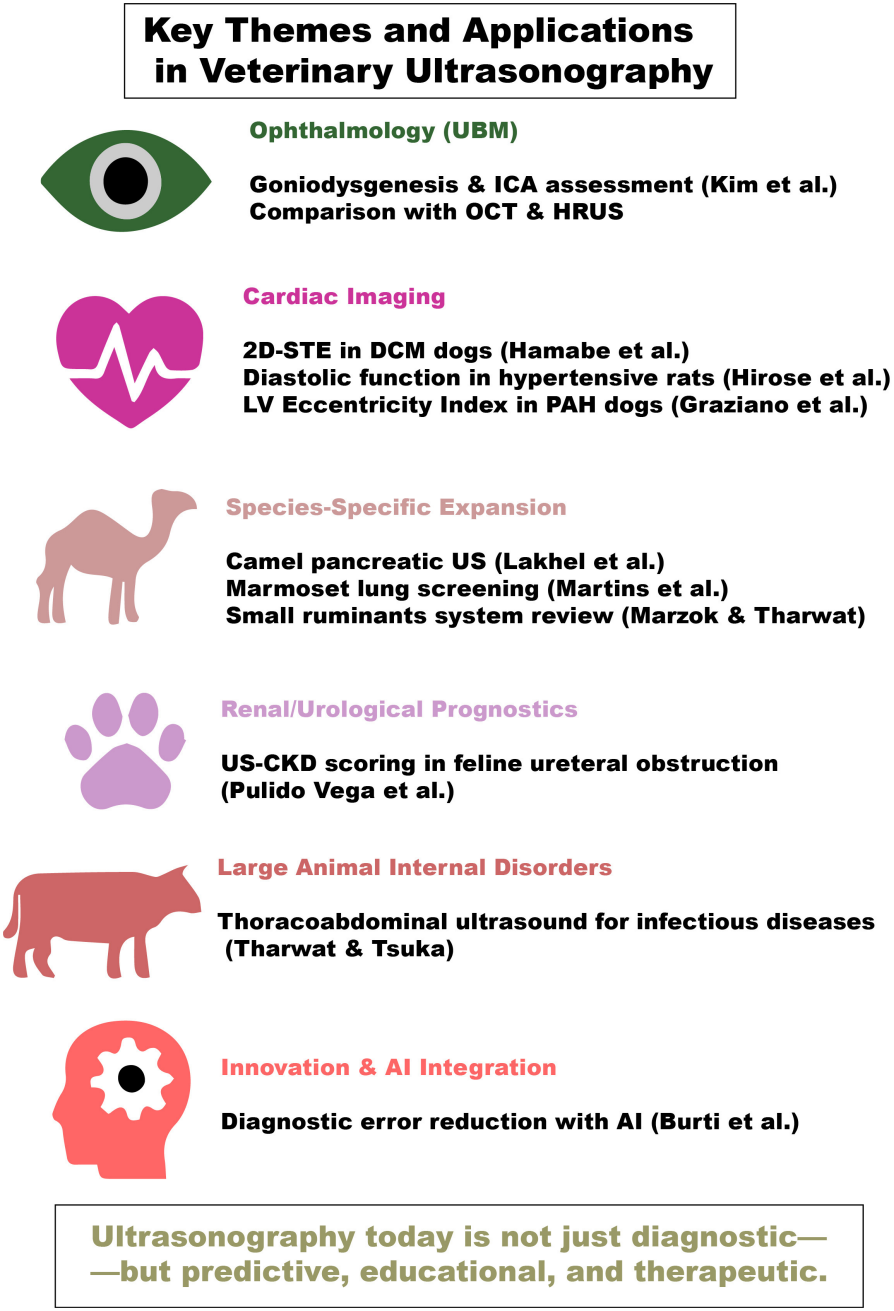
Schematic presentation of the outstanding applications of ultrasonography in veterinary practice.

Veterinary diagnostic ultrasonography has long stood as a cornerstone of modern clinical veterinary medicine. Its non-invasive nature, real-time imaging capabilities, and evolving technological sophistication have positioned it as an indispensable tool across species and disciplines. In recent years, advances in ultrasonographic techniques have opened new frontiers, not only in diagnosis but also in disease monitoring, treatment planning, and prognostics (1). Furthermore, ultrasonography could be used efficiently to provide qualitative evaluation and quantitative scoring of the healing following surgeries (2). In addition, color Doppler ultrasonography has been utilized to assess the function of different organs through evaluation of their vascular hemodynamics. Engagement of novel technologies in ultrasonography presented a novel milestone that extended the uses of ultrasonography to early detection and even prediction of many cardiac diseases. That consequently aids in designing and assessing prompt therapeutic protocols and minimizes the possible deaths (3–5). The present Research Topic, *Outstanding Advances in Veterinary Diagnostic Ultrasonography: Novel Milestones in Disease Detection, Prediction, and Treatment*, brings together groundbreaking research that highlights the most innovative applications and conceptual developments in this domain (Figure 1).

The 13 studies collected here illustrate the diversity and dynamic nature of ultrasonography in the veterinary field. These studies span a range of species, including companion animals, livestock, and exotic species, and encompass clinical, translational, and methodological perspectives. A common theme that emerges is the integration of ultrasonography with other diagnostic and computational technologies, marking a shift toward more predictive and individualized veterinary care.

One of the prominent themes across the collected works is the refinement of cardiac imaging. Hamabe et al. presented a compelling application of two-dimensional speckle-tracking echocardiography (2D-STE) in retrievers with dilated cardiomyopathy (DCM), demonstrating that myocardial strain imaging enables earlier and more sensitive detection of ventricular dysfunction than conventional methods. Similarly, Hirose et al. explored diastolic dysfunction using color M-mode Doppler imaging in hypertensive rat models, highlighting how intraventricular pressure gradients (IVPGs) are influenced by mitral inflow patterns and heart rate findings that hold translational value for both experimental and clinical cardiology.

The utility of ultrasonography in pulmonary vascular disease was examined in depth by Graziano et al. who evaluated the left ventricular eccentricity index (EI) as a non-invasive measure to detect moderate-to-severe precapillary pulmonary hypertension (PAH) in dogs. Their multicenter study showed strong inter-observer agreement and provided validated cut-off values, establishing EI as a valuable tool in emergency and critical care settings where rapid point-of-care assessments are critical.

In the realm of nephrology and internal medicine, Pulido Vega et al. investigated contralateral kidney ultrasonographic scoring in cats with unilateral ureteral obstruction. Higher US-CKD scores in the non-obstructed kidney were found to be predictive of poor long-term renal function, demonstrating that structured ultrasonographic evaluation may help inform prognosis and therapeutic strategy in feline urological emergencies.

Ophthalmologic ultrasonography, particularly ultrasound biomicroscopy (UBM), was explored in two complementary articles. First, Kim et al. quantitatively assessed the ciliary cleft and iridocorneal angle in dogs with primary glaucoma and goniodysgenesis, revealing structural predictors of disease. In the second study, Kim et al. outlined the technical advantages of UBM over other imaging modalities such as optical coherence tomography (OCT) and high-resolution ultrasound, supporting UBM's growing role in anterior segment evaluation and screening in canine ophthalmology.

Beyond companion animals, several studies in this Research Topic illustrate the expanding scope of ultrasonography in livestock and exotic species. Lakhel et al. pioneered an ultrasonographic evaluation of the pancreas in dromedary camels, identifying characteristic echogenic patterns across anatomical regions and age groups. Meanwhile, Martins et al. reported on thoracic ultrasound findings in neotropical primates (*Callithrix* spp.), demonstrating that subclinical pneumopathies could be identified using ultrasonography, an important step forward in wildlife medicine and conservation diagnostics.

Tharwat and Tsuka reviewed ultrasonographic features of bacterial and parasitic diseases in ruminants, providing a detailed imaging atlas of thoracic and abdominal pathologies, including reticuloperitonitis and pleuropneumonia. Their work underscores the continuing value of ultrasound in field-based large animal diagnostics. Along similar lines, Marzok and Tharwat presented an illustrated guide to ultrasonographic findings in small ruminants, covering diseases of the digestive, respiratory, urinary, and cardiovascular systems—a practical reference for practitioners in production animal medicine.

Among the technical and clinical innovations highlighted in this Research Topic, the study by Saito et al. offers valuable insights into the therapeutic monitoring of cardiac disease in cats. By evaluating myocardial function before and after carvedilol administration in cats with obstructive hypertrophic cardiomyopathy (HOCM), the authors demonstrated improvements in echocardiographic parameters, including left ventricular fractional shortening and myocardial strain. These findings suggest that ultrasonography can serve not only as a diagnostic tool but also as a non-invasive means of evaluating treatment efficacy and guiding long-term management strategies in feline cardiology.

Education and training in ultrasonography are also undergoing transformation. Guillaumin et al. assessed a hybrid online/in-person POCUS (point-of-care ultrasound) course targeting early-career emergency veterinarians. Their findings indicate that targeted training significantly improves anatomical recognition and confidence in ultrasonographic procedures, supporting the implementation of structured POCUS education in veterinary curricula and continuing professional development.

Finally, the potential of artificial intelligence in veterinary imaging was explored by Burti et al. in their perspective on AI integration for error reduction in diagnostic interpretation. The article not only outlines the challenges of standardization and validation in machine learning but also provides a forward-thinking roadmap for incorporating AI into routine veterinary workflows, including telemedicine and automated image analysis.

Taken together, the articles in this Research Topic present a cohesive and forward-looking view of ultrasonography as a central pillar of veterinary diagnostics. They demonstrate that ultrasound is no longer simply a tool for detection but a predictive, educational, and even interventional platform. By linking imaging findings to outcomes, leveraging emerging technologies, and adapting techniques to underrepresented species and settings, the field is evolving toward a more comprehensive, and integrative diagnostic model.

The road ahead is promising. The continued incorporation of ultrasonography into artificial intelligence systems, point-of-care decision-making tools, and species-specific protocols will further enhance its clinical impact. Equally important is the expansion of ultrasonographic training and credentialing to ensure consistency and competency among practitioners at all levels. As demonstrated by the works in this Research Topic, veterinary ultrasonography is not only keeping pace with modern veterinary practice—it is actively shaping its future.
